# Antioxidant Responses Induced by UVB Radiation in *Deschampsia antarctica* Desv.

**DOI:** 10.3389/fpls.2017.00921

**Published:** 2017-05-31

**Authors:** Hans Köhler, Rodrigo A. Contreras, Marisol Pizarro, Rodrigo Cortés-Antíquera, Gustavo E. Zúñiga

**Affiliations:** Laboratorio de Fisiología y Biotecnología Vegetal, Departamento de Biología, Facultad de Química y Biología – Centro para el Desarrollo de la Nanociencia y Nanotecnología, Universidad de Santiago de ChileSantiago, Chile

**Keywords:** *Deschampsia Antarctica* Desv., Poaceae, Antarctica, UVB radiation, antioxidant responses

## Abstract

*Deschampsia antarctica* Desv. is one of two vascular plants that live in the Maritime Antarctic Territory and is exposed to high levels of ultraviolet-B (UVB) radiation. In this work, antioxidant physiology of *D. antarctica* was studied in response to UVB induced oxidative changes. Samples were collected from Antarctica and maintained *in vitro* culture during 2 years. Plants were sub-cultured in a hydroponic system and exposed to 21.4 kJ m^-2^ day^-1^, emulating summer Antarctic conditions. Results showed rapid and significant increases in reactive oxygen species (ROS) at 3 h, which rapidly decreased. No dramatic changes were observed in photosynthetic efficiency, chlorophyll content, and level of thiobarbituric acid reactive species (MDA). The enzymatic (superoxide dismutase, SOD and total peroxidases, POD) and non-enzymatic antioxidant activity (total phenolic) increased significantly in response to UVB treatment. These findings suggest that tolerance of *D. antarctica* to UVB radiation could be attributed to its ability to activate both enzymatic and non-enzymatic antioxidant systems.

## Introduction

Since the 1980s, stratospheric ozone has been catalytically broken down by the introduction of man-made chlorofluorocarbon compounds into the atmosphere, resulting in significantly decreased ozone levels (Solomon, 2004; Shanklin, 2010). The largest extent of this depletion occurs during the austral spring when an ozone hole forms above Antarctica. This consequently decreases the shielding effect of the ozone layer in this region which results in an increase in ultraviolet-B radiation (UVB; 280–315 nm) reaching the surface of Antarctica (Kerr and McElroy, 1993; Ruhland and Day, 2000). Although ozone depletion models predict slow Antarctic ozone recovery, the seasonal and long-term levels of damaging UVB radiation are likely to remain high for decades to come (Kramarova et al., 2014; Solomon et al., 2016).

The most common effects of UVB radiation on plant physiology are reduced biomass (Tevini and Teramura, 1989), alteration of the cuticle and the epidermis (Tevini and Steinmüller, 1987), abnormal growth and impaired photosynthesis (Teramura et al., 1990; Teramura and Sullivan, 1994), and damage to the photosystem I (PSI) and photosystem II (PSII) proteins (Pang and Hays, 1991; Liu et al., 2013). At the cellular level, UVB radiation initially causes an increase in reactive oxygen species (ROS) levels, which subsequently oxidizes proteins, lipids, and other biomolecules, thus, compromising the functionality and integrity of enzymes and cell membranes (Kochevar, 1990; Robson et al., 2015). Two ROS scavenging mechanisms can control this oxidative stress in plants: enzymatic and non-enzymatic antioxidant systems (Mittler, 2002).

Firstly, antioxidant enzymes act constantly to control and detoxify ROS, increasing their activity in response to high levels of oxidative species (Mittler, 2002). An important antioxidant enzyme is the superoxide dismutase (SOD, EC 1.15.1.1), a metalloenzyme that plays a key role in protecting molecules in plants from oxidation damage. SOD functions by dismutating the superoxide ion (O_2_^-^) into less harmful hydrogen peroxide (H_2_O_2_), which can then be converted to H_2_O by ascorbate peroxidase (APX, EC 1.11.1.11), total peroxidases (POD) and catalase (CAT, EC 1.11.1.6) enzymes. These enzymes work together in the regulation of ROS levels through their role in the water–water cycle and water–ascorbate–glutathione cycle (Mittler, 2002).

Secondly, detoxification and regulation of ROS through non-enzymatic mechanisms involve secondary metabolites, compounds that plants produce in response to several environmental conditions (Dixon and Paiva, 1995). These metabolites include types of phenolic compounds, flavonoids, and hydroxycinnamic acid esters; most of these compounds have antioxidant activity, i.e., the ability to scavenge free radicals. These secondary metabolites are not only essential for their antioxidant properties, because they also directly absorb UVB wavelengths, acting potentially as sunscreens forming part of a common protection mechanism in plants (Frohnmeyer and Staiger, 2003; Petersen et al., 2010). The biosynthesis of secondary metabolites is highly regulated by the phenylalanine ammonia lyase (PAL) (E.C. 4.1.1.5) (Winkel-Shirley, 2002), a key enzyme in the response against adverse environmental conditions such as UVB radiation (Kalbin et al., 2001). An increase in UVB light enhances PAL activity which increases the production of phenolic metabolites that directly and indirectly protect against UVB-induced damage (Hideg et al., 2013).

Fluctuations in UVB radiation represent an ongoing stress to plants. For example, in Tierra del Fuego, in southern South America, native plants show limited or no acclimation responses to environmental UVB radiation, such as an increasing of sunscreen compounds levels or an enhanced DNA repair capacity (Sancar and Sancar, 1988; Rousseaux et al., 1999). In sub-Antarctic regions, the impact of short periods of increased UVB radiation is related to an increase of DNA damage levels in leaf tissue in the native herb *Gunnera magellanica* (Rousseaux et al., 2004).

Antarctica represents an extreme environment, the presence of high soil salinity, low water potential, low temperatures, and drastic changes in white light and UVB radiation levels in the transition from winter (absence of light) to spring–summer (high light intensities). It has been reported that extreme shifts in UVB light occur not only over decades, but there are also annual, monthly, and daily variations that induce significant changes at different levels in Antarctic plants (Xiong and Day, 2001; Robinson et al., 2003). During the summer growing period (November to February), the day length is approximately 20 h, and the white light irradiation in the maritime Antarctic can exceed levels of 1600 μmol m^-2^ s^-1^ (Xiong and Day, 2001; Rozema et al., 2002), so these plants are subjected to contrasting environmental variations daily.

Daily and seasonal variation in UVB radiation induces antioxidant responses, including increases in protective secondary metabolite synthesis, in Antarctic vegetation. Despite extreme conditions, *D. antarctica* is the most successful angiosperm that has colonized the maritime Antarctic territory (Robinson et al., 2003; Hyoungseok et al., 2008). It has been suggested that tolerance of *D. antarctica* to changing levels of UVB radiation *in situ* is due to its synthesis and storage of phenolic-type molecules that play an important role in protecting biomolecules (Xiong and Day, 2001; van de Staaij et al., 2002). This response was observed when this Antarctic hairgrass was exposed to UVB radiation under controlled conditions in a photobioreactor (Sequeida et al., 2012).

Despite the relevant role of antioxidant responses in its UVB radiation tolerance of *D. antarctica*, the specific antioxidant mechanisms involved in its capacity to survive in high UVB light environments remain uncharacterized. Therefore, the aims of this work were to determine the effect of UVB radiation on various physiological parameters and to monitor several enzymatic and non-enzymatic antioxidant responses of *D. antarctica* exposed to daily UVB radiation doses equivalent to Antarctic conditions. We hypothesized that *D. antarctica* utilizes many efficient antioxidant mechanisms in order to survive under elevated UVB radiation due to ozone depletion.

## Materials and Methods

### Plant Material

**FIGURE 1:**
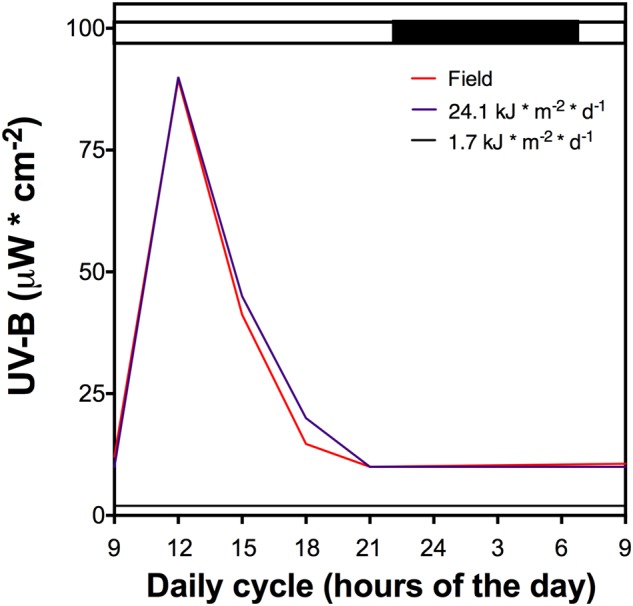
The UV-B light cycle that the cultured *D. antarctica* plants were exposed, purple line represents 21.4 kJ m^-2^ d^-1^ dose, black line the control (1.7 kJ m^-2^ d^-1^) and red line UV-B data recorded in field conditions. Light period is indicated by a white bar and darkness by a black bar above the figure. The dark period shows a low UV-B radiation dose.

Whole plants of *D. antarctica* were collected from King George Island, South Shetland Islands (62°814′S; 58°848′W) and cultured *in vitro* (Zamora et al., 2010). Plants were cultured in a growth chamber (16 ± 2°C, 16/8-h light/dark period) under a low UVB radiation dose (1.7 kJ m^-2^ d^-1^, growth chamber) (**Figure 1**). Plantlets were acclimated in an *ex vitro* culture in hydroponic conditions using Hoagland No. 2 solution (Hoagland and Arnon, 1950) in volcanic pearl for 2 weeks under the low UVB radiation dose described above. Afterward, cultured plants underwent a UVB light treatment that was performed using mercury lamps (Supplementary Figure 1) and a total daily dose of 21.4 kJ m^-2^ d^-1^ (**Figure 1**), similar to field UVB doses measured in King George Island (unpublished data). Control plants were exposed to a white lamp with a residual UVB radiation dose of 1.7 kJ m^-2^ d^-1^. All control and UVB treated samples were collected and maintained at -80°C before processing.

### Total Reactive Oxygen Species (ROS)

Total ROS was analyzed using fluorimetric quantitation of dichlorodihydrofluorescein-diacetate (DCDHF-DA) that was oxidized by ROS. Fresh plant tissue (100 mg) was incubated in 1 mL of 10 μM of DCDHF-DA in Tris-HCl (50 mM, pH 8.0) for 1 h at room temperature, then the tissue was washed with 50 mM EDTA to remove the incubation solution and ground to a fine powder as above and extracted in 1 mL of Tris-HCl (50 mM, pH 8.0). The supernatant obtained from this mixture after centrifugation at 10,000 rpm for 10 min was filtered in Miracloth. The fluorescence (488 nm excitation wavelength, 535 nm emission wavelength; Perkin-Elmer, LS4) was determined for the filtered supernatant (Ross et al., 2008).

### Membrane Peroxidation

Fresh tissue (50 mg) was ground to a powder as above and suspended in 1 mL of 1% of trichloroacetic acid (TCA). The resultant mixture was centrifuged at 8,000 rpm for 5 min. To the supernatant (250 μL), 1 mL of 0.5% of thiobarbituric acid in 20% TCA was added and the mixture was boiled for 30 min. This was then allowed to cool to room temperature and the adduct formed by TBA-malondialdehyde (MDA) was quantified at 532 and 600 nm using 𝜀 = 155 mM^-1^ cm^-1^ (Ederli et al., 2004).

### Photosynthetic Pigments (Chl-*a/b*)

The total content of Chl-*a* and Chl-*b* was determined using fresh tissue (100 mg) that was ground to a powder using liquid nitrogen and a mortar and pestle before extraction in acetone (10 mL). The mixture was centrifuged at 8,000 rpm (Biofuge Fresco, Heraeus Inst., Hanau, Germany) for 15 min at 4°C and the absorbance of the supernatant at 649 and 665 nm was measured on a UV-Vis spectrophotometer (Agilent 8453, Santa Clara, CA, United States) (Lichtenthaler and Wellburn, 1983).

### Photosynthetic Efficiency

A photosynthetic efficiency analyzer (PEA; Hansatech, Norfolk, United Kingdom) was used to measure photosynthetic efficiency. Etiolating clamps were placed on the leaves for 30 min and the variable and maximum fluorescence of PSII was measured. Results are expressed as *F*v/*F*m (PSII maximum efficiency).

### Antioxidant Enzymes Extraction

Fresh tissue (100 mg) was ground to a fine powder and extracted in 1 mL of sodium phosphate buffer (50 mM, pH 7.5). Mixture was centrifuged at 10,000 rpm for 10 min at 4°C. Supernatant was recovered and the concentration of the soluble proteins was determined according to Bradford methodology (Bradford, 1976) using bovine serum albumin (BSA) for the standard curve. This extract is referred to as the protein extract for the following enzyme activity assays.

### Superoxide Dismutase (SOD) Activity (EC 1.15.1.1)

A reaction mixture was prepared using 600 μL of sodium phosphate buffer (50 mM, pH 7.5), 10 μL of 10 mM EDTA, 100 μL of 130 mM methionine, 10 μL of 2 mM riboflavin, 200 μL of 3 mM of nitroblue tetrazolium in 70% dimethylformamide and 100 μL of protein extract. The mixture was incubated under white light for 15 min at room temperature (a blank mixture was kept in the dark). Absorbance at 560 nm was determined where one enzymatic unit (EU) was considered to have the capacity to inhibit 50% of photochemical reduction of NBT (Beauchamp and Fridovich, 1971).

### Ascorbate Peroxidase (APX) Activity (EC 1.11.1.11)

A reaction mixture that contained 935 μL of sodium phosphate buffer (50 mM, pH 7.5), 20 μL of protein extract, 5 μL of 100 vol. hydrogen peroxide and 40 μL of 10 mM sodium ascorbate was prepared. Absorbance at 290 nm was recorded after the reaction had proceeded for 1 min indicating APX activity in terms of ascorbate consumption. APX activity was calculated using molar extinction of ascorbate, 𝜀 = 2.8 mM^-1^ cm^-1^ (Lima et al., 2002).

### Total Peroxidases (POD) Activity (EC 1.11.1.7)

A reaction mixture that contained 980 μL of sodium phosphate buffer (50 mM, pH 7.5), 10 μL of protein extract, 5 μL of 100 vol. hydrogen peroxide and 5 μL of guaiacol was prepared. Absorbance at 470 nm was recorded after a reaction time of 1 min indicating POD activity in terms of tetrahydroguaiacol (THG) formation. POD activity was calculated using molar extinction of THG, 𝜀 = 26.6 mM^-1^ cm^-1^ (Zamora et al., 2010).

### Catalase (CAT) Activity (EC 1.11.1.6)

A reaction mixture that contained 975 μL of sodium phosphate buffer (50 mM, pH 7.5), 20 μL of protein extract and 5 μL of 100 vol. hydrogen peroxide was prepared. Absorbance at 240 nm was recorded after reaction time of 1 min indicating CAT activity in terms of hydrogen peroxide consumption. CAT activity was calculated using molar extinction of hydrogen peroxide, 𝜀 = 39.4 mM^-1^ cm^-1^ (Lima et al., 2002).

### Phenylalanine Ammonia Lyase (PAL) Activity (EC 4.3.1.24)

Proteins were extracted from ground fresh tissue (100 mg) using x mL of 50 mM of Tris-HCl (pH 8.5) containing 14.4 mM of 2-mercaptoethanol and 5% PVPP-40. After the mixture was centrifuged at 11,000 rpm for 10 min, total protein content of the supernatant was analyzed using the Bradford method (Bradford, 1976). To measure PAL activity, two different mixtures were prepared: one containing 2.5 mL of 0.2% L-phe and 500 μL of protein extract; and another containing 0.2% D-phe in Tris-HCl (pH 8.5), which was used as the negative control. The sample and control mixtures were incubated at 38°C for 2 h, allowing the reaction to proceed. After incubation, the *trans-*cinnamic acid product was detected at 290 nm. The difference between L-phe and D-phe was used to calculate the activity (Pellegrini et al., 1994).

### Plant Hydroalcoholic Extracts

A total of 100 mg of fresh plant material was mixed with 1 mL of ethanol (85% v/v) and sonicated at 50–60 Hz for 2 h at 25°C according to the method previously described by Contreras et al. (2015). Extracts were filtered in a 0.45 μm pore filter (Millipore, Billerica, MA, United States) and analyzed for total phenolic content.

### Total Phenolic Content

The total phenolic content was determined using a modified Folin-Ciocalteu colorimetric method (Contreras et al., 2015). Plant hydroalcoholic extract (40 μL) was added to 100 μL of Folin-Ciocalteu’s reagent and 560 μL of deionized water and mixed. After 15 min at room temperature, the reaction was stopped by adding 300 μL of 7% aqueous sodium carbonate to the mixture. The absorbance was measured at 660 nm on an Agilent 8453 UV-Vis spectrophotometer. The results were expressed in gallic acid (GA) equivalents per gram of DW.

### Non-enzymatic Antioxidant Scavenging Activity

The 1,1-diphenyl-2-picrylhydrazyl (DPPH) free-radical scavenging assay was used to measure the capacity of non-enzyme compounds to scavenge free radicals (Naik et al., 2005). Plant hydroethanolic (85%) extract (100 μL) was added to 900 μL of DPPH (A_517_ = 0.75) and the absorbance at 517 nm was measured after a 5 min incubation at 37°C. Antioxidant activity is expressed as percentage DPPH consumption where DPPH (A_517_ = 0.75) was used as a control reference (Naik et al., 2005).

### The Antioxidant Activity Assay/Reducing Power Assay (FRAP)

The antioxidant activity assay/reducing power assay (FRAP) was performed using the method described in Benzie and Strain (1999). Absorbance kinetics (4 min) were determined at 593 nm on a spectrophotometer. The results are expressed as Trolox equivalents (E-Trolox).

### Statistical Analysis

All the experiments were performed in triplicates, for statistically significant differences we used two-way ANOVA with multiple comparisons. *Post hoc* analysis was performed using the Bonferroni’s post-test (*P* < 0.05).

## Results

**FIGURE 2:**
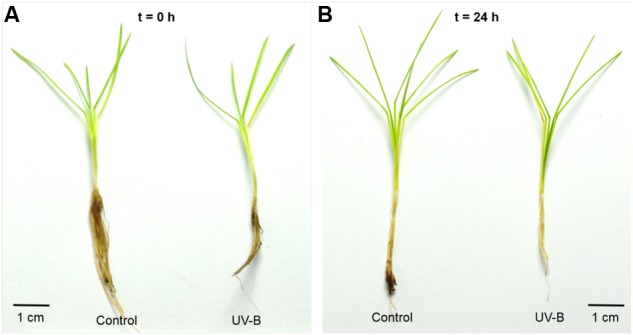
Photographs of *D. antarctica* plants that were treated to a 1 day cycle of UVB radiation (UVB) in comparison with the control plants (Control) at the beginning of the experiment (**A**; *t* = 0 h) and after 24 h (**B**; *t* = 24 h). The scale bar indicates a length of 1 cm.

**FIGURE 3:**
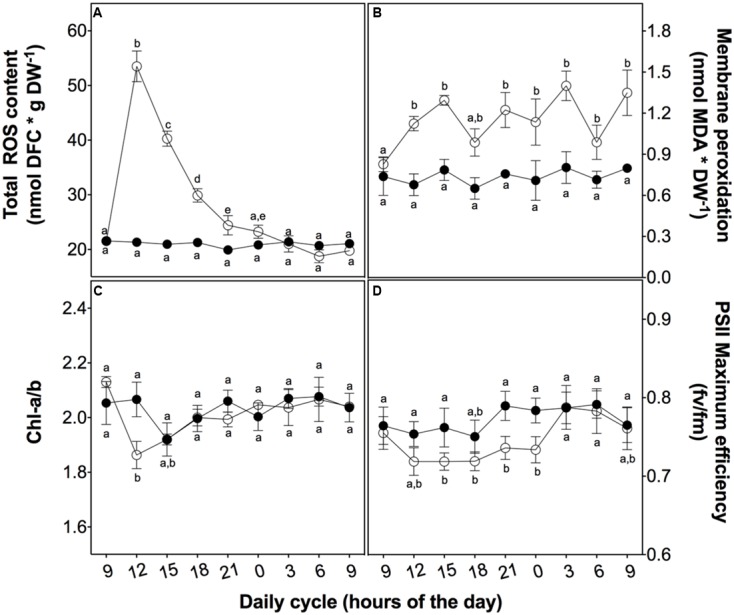
Effect of UVB radiation on physiological parameters in *D. antarctica* plants grown *ex vitro*. **(A)** Total ROS content. **(B)** Membrane peroxidation (thiobarbituric acid reactive substances). **(C)** The ratio of chlorophyll *a* and *b*. **(D)** Photosynthetic efficiency of PSII. Open circles represent means (±standard error of the mean; *N* = 3) of plants that underwent the UVB treatment and closed circles represent the control means (±standard error of the mean; *N* = 3). Significant differences between treatments are indicated by letters (^∗^*P* < 0.05).

After a daily dose of UVB (21.4 kJ m^-2^ d^-1^), *D. antarctica* plants showed a similar appearance compared to the control (**Figure 2**). However, there were biochemical and physiological differences found in response to UVB radiation (**Figure 3**). Total ROS content and membrane peroxidation showed a maximum peak of accumulation at 3 h of UVB exposure before gradually declining to initial levels over the next 12 h (**Figure 3A**). ROS levels in the control plants did not change significantly over the day experiment. Membrane peroxidation content remained consistently and significantly higher in the UVB treated plants compared with the control plants (**Figure 3B**). In contrast, the chlorophyll *a*/*b* ratio presented a temporary decrease of 9.8% with respect to the control after 3 h of UVB exposure (**Figure 3C**). The PSII maximum efficiency was slightly affected by the treatment in values nearby to 5% (**Figure 3D**). These results suggest that *D. antarctica* may control ROS to maintain the cell function.

**FIGURE 4:**
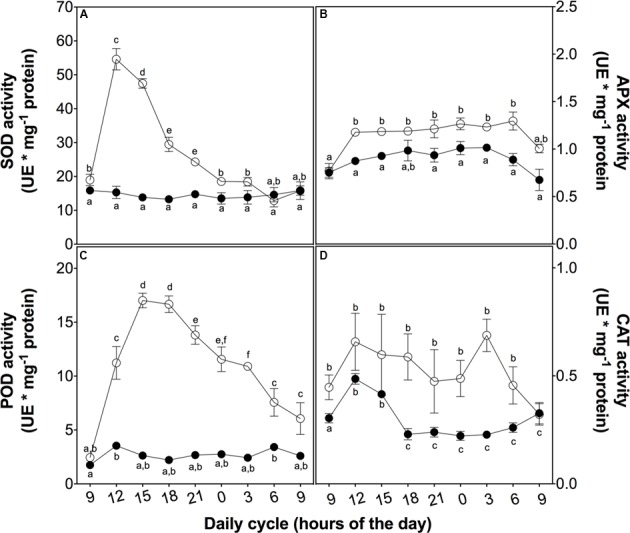
Antioxidant enzyme activity *in D. antarctica* plants exposed to UVB radiation and control conditions. Superoxide dismutase activity **(A)**, ascorbate peroxidase activity **(B)**, the total activity of peroxidases **(C)** and catalase activity **(D)** is observed. Open circles represent means (±standard error of the mean; *N* = 3) of plants that underwent the UVB treatment and closed circles represent the control means (±standard error of the mean; *N* = 3). Significant differences between treatments are indicated by letters (^∗^*P* < 0.05).

To evaluate the mechanisms involved in the ROS buffering, we analyzed the activity of both enzymatic and non-enzymatic antioxidant systems. At 3 h of UVB exposure, a peak in SOD activity associated with the ROS content peak was observed. Both of these parameters then decreased in a similar way (**Figure 4A**). APX activity also showed a slight increase at 3 h of treatment but this higher level was maintained until the end of the cycle (**Figure 4B**), similar to membrane peroxidation levels and loss of PSII maximum efficiency (**Figures 3B,D**). POD activity rapidly increased in the UVB treated plant in the first 3 h of UVB exposure reaching a level ten times than the control plants after 6 h (**Figure 4C**). Although CAT showed an upregulation in UVB treatment, the activity was more discrete compared with the other antioxidant enzymes measured (**Figure 4D**). These results suggest that POD is the principal H_2_O_2_ scavenger enzyme.

**FIGURE 5:**
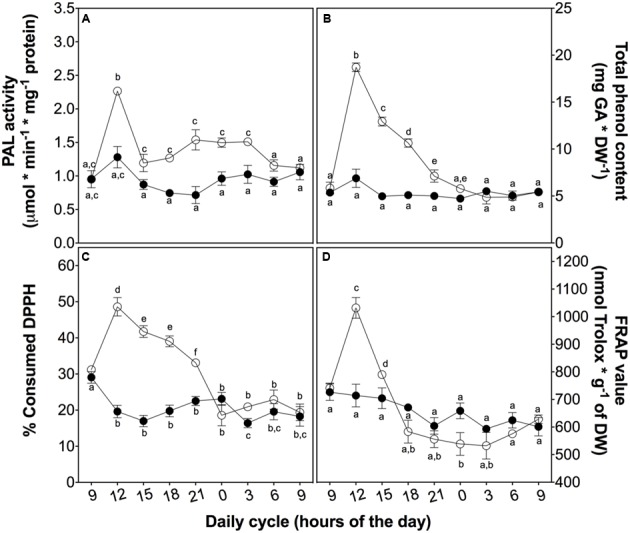
Non-enzymatic antioxidant activity in *D. antarctica* plants exposed to UVB radiation and control conditions. PAL activity **(A)** The total phenolic content **(B)**, the consumption of DPPH radical **(C)** and reducing power of hydroalcoholic extracts **(D)** is observed. Open circles represent means (±standard error of the mean; *N* = 3) of plants that underwent the UVB treatment and closed circles represent the control means (±standard error of the mean; *N* = 3). Significant differences between treatments are indicated by letters (^∗^*P* < 0.05).

To investigate the role of phenolic compounds as non-enzymatic antioxidants, we measured the total content of phenolic compounds and their antioxidant activity as a reductive power and free radical-scavenger (**Figure 5**). Firstly, the activity of the key enzyme of the phenylpropanoid metabolism, PAL, peaked at 3 h in treated plants (**Figure 5A**). This peak is likely associated with total phenolic content (**Figure 5B**), ROS levels (**Figure 3A**), and SOD and POD activities (**Figures 4A,C**), suggesting a complementary role of phenolic compounds as a H_2_O_2_ controller.

Secondly, to explain the role of phenolics, we evaluated the free radical-scavenging activity and the reducing power of *D. antarctica* secondary metabolites. DPPH-scavenging and reducing power (**Figures 5C,D**) showed a peak of activity at 3 h, strictly related to other antioxidant parameters, such as those mentioned above, supporting the hypothesis of the ROS buffering role of phenolics.

## Discussion

Plants respond to UVB radiation through a series of strategies and mechanisms (Müller-Xing et al., 2014). Under UVB radiation, leaf morphology changes and presents a curly arrangement, but in this study *D. antarctica* leaves showed no apparent morphological damage when exposed to a daily UVB dose of 21.4 kJ m^-2^ day^-1^. This lack of morphological changes monitored over a 24-h period does not mean a lack of response against this condition (Rao et al., 1996).

Reactive oxygen species accumulation is largely described as a stress marker in aerobic organisms; in plants many authors have described its accumulation in response to environmental changes, including UVB radiation. Here, we show that total ROS levels in *D. antarctica* sharply increased after 3 h of UVB exposure and then gradually declined. We expected to observe an effect of this climax in ROS levels in the generation of lipoperoxides as membranes are one of the most susceptible targets to ROS accumulation. In the case of *D. antarctica*, significantly higher levels of lipoperoxides were found in the UVB treated plants, but these were quite low especially when compared with other plant species, such as rice (Du et al., 2010).

In contrast, the chlorophyll *a*/*b* ratio showed a decrease in plants at 3 h of UVB exposure compared to the control plants. This observation was expected because pigments of the photosynthetic apparatus are negatively affected by UVB radiation (Jansen et al., 1998; Vass et al., 2005). Chlorophyll pigments and some proteins that are components of PSI and PSII are frequently oxidized through changes in light intensity (Von Wettstein et al., 1995; Chatterjee and Kundu, 2015). UVB light not only disturbs chlorophyll synthesis, it also affects photosynthetic efficiency of PSII (Edelman and Mattoo, 2008) and subsequently alters the photosynthetic efficiency (Heifetz et al., 1997).

Ultraviolet-B treated *D. antarctica* showed a discrete but significant decrease in *F*v/*F*m, revealing a partial stress (a decrease of 5% relative to the control plants), and it recuperated to normal levels after 3 h in the dark, supporting the efficiency of stress responsive elements (Byun et al., 2015). This is probably because this particular Antarctic plant has developed several mechanisms to dissipate excess energy reaching chloroplasts (Ruhland and Day, 2000; Frohnmeyer and Staiger, 2003; Ruhland et al., 2005) Effects. *D. antarctica* has not shown signs of photoinhibition when exposed to bright light and neither have thermal deactivation mechanisms been detected (Pérez-Torres et al., 2007). No evidence of dramatic loss of photosynthetic efficiency was observed, suggesting that *D. antarctica* utilizes active response mechanisms that were previously unknown (Ruhland and Day, 2000; Pérez-Torres et al., 2007).

Controlling oxidative damage is a trait that depends on the plasticity of a species that allows a rapid response to environmental conditions (Smith, 1990; Tognetti et al., 2012). Our results show a plastic response of *D. antarctica* to a UVB-induced increase of ROS levels by initiating a series of enzymatic and non-enzymatic processes to prevent significant damage to plasma membranes or other organelles (Foyer and Noctor, 2005; Pereira et al., 2009). These processes involved SOD and POD enzymes and the secondary phenolic metabolites produced by the Antarctic hairgrass.

According to the results, *D. antarctica* showed efficient SOD and POD activities, complemented with a significant increase in antioxidant phenolics. SOD is known to be the enzyme that dismutates O_2_^-^ to H_2_O_2_ and probably acts in a complex of antioxidant enzymes involved in signal transduction (Xia et al., 2015). On the other hand, H_2_O_2_ acts as a secondary messenger promoting antioxidative responses, i.e., increase of endogenous H_2_O_2_ is related to acclimation to stress in rice (Orozco-Cárdenas et al., 2001; Uchida et al., 2002).

Downstream from SOD action, H_2_O_2_ scavenging enzymes like class I PODs, such as APX, or class III PODs have different catalytic mechanisms (De Gara, 2004). Unlike APX, POD does not use ascorbate as a direct electron donor; however, POD has the property of oxidizing other molecules, especially natural phenolic compounds (De Gara, 2004). This, in addition to the increase in the total content of phenolic molecules, may explain the slight increase of APX activity compared to the high activity of POD. Class III PODs are found in various cellular compartments, including apoplasts, and have a wide range of isoforms, properties that allow POD to be rapidly activated by various stresses and highly efficient in the metabolism of H_2_O_2_. Furthermore, these enzymes are considered as an indicator of stress responses and might allow cellular responses to evaluate the intensity of an adverse environmental condition (Blokhina et al., 2003; Suzuki et al., 2012). Therefore, both SOD and POD playing important and effective roles in the enzymatic response of *D. antarctica* to high levels of UVB-induced ROS.

Enzymatic ROS scavenger mechanism is also complemented by a non-enzymatic antioxidant system response in *D. antarctica*, which is evident by the significant increases of total phenolics and bulk antioxidant activity after 3 h of exposure to UVB light. Some authors claim that the UVB tolerance of this plant is due to chemical properties these metabolites (Ruhland and Day, 2000; Xiong and Day, 2001). We showed a peak in total phenolic content of *D. antarctica* at 3 h of exposure, which related to percentage of consumed DPPH and FRAP values, suggesting that these phenolics function as soluble antioxidant metabolites. In terms of regulation, the PAL activity supports the hypothesis of *de novo* biosynthesis induced by UVB radiation in *D. antarctica* (Jaakola and Hohtola, 2010; Hideg et al., 2013). Thus, these secondary metabolites are not only important for their high antioxidant activity, but also for their photoprotective properties (Rozema et al., 2002; Winkel-Shirley, 2002; Pereira et al., 2009).

## Conclusion

Our results suggest that (i) *Deschampsia antarctica* Desv. employs both enzymatic and non-enzymatic antioxidant systems that act as a whole to protect this Antarctic monocot against oxidative stress when exposed to natural daily fluctuations in UVB radiation *ex vitro*, (ii) the rapid activation of these mechanisms in this Antarctic hairgrass may explain its high tolerance to changes in UVB light in Antarctica. Our results support the hypothesis that antioxidant systems mediate the capacity of *D. antarctica* to survive in UVB enriched environments.

Further investigations need to focus on determining the role of antioxidant systems in the accumulative effect of UVB radiation in *D. antarctica* in field conditions.

## Author Contributions

HK designed and performed the experiments, and was the main contributor in writing the manuscript. RC designed and performed the experiments, produced the figures and performed the statistical analyses. MP and RC-A performed some experiments. GZ designed experiments, supervised all experiments and edited the manuscript.

## Conflict of Interest Statement

The authors declare that the research was conducted in the absence of any commercial or financial relationships that could be construed as a potential conflict of interest.
