# Supplementary material for: Antioxidant Responses Induced by UVB Radiation in Deschampsia antarctica Desv
Source: Front Plant Sci. 2017 May 31;8:921. doi: 10.3389/fpls.2017.00921 (PMC5449467; doi:10.3389/fpls.2017.00921)
Supplement: Supplementary file 1 [file Data_Sheet_1.docx]

Supplementary figure


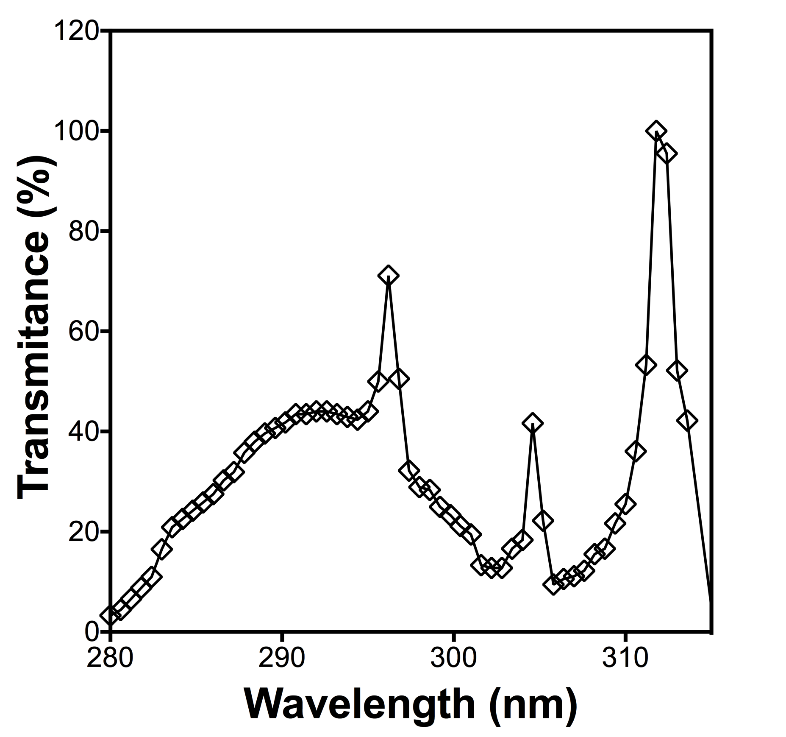


Supplementary figure 1. Emission spectra of UVB lamps used in experimental design.
